# Lymphatic filariasis patient identification in a large urban area of Tanzania: An application of a community-led mHealth system

**DOI:** 10.1371/journal.pntd.0005748

**Published:** 2017-07-14

**Authors:** Upendo Mwingira, Maria Chikawe, Wilfred Lazarus Mandara, Hayley E. Mableson, Cecilia Uisso, Irene Mremi, Alpha Malishee, Mwele Malecela, Charles D. Mackenzie, Louise A. Kelly-Hope, Michelle C. Stanton

**Affiliations:** 1 Neglected Tropical Diseases Control Programme, Ministry of Health and Social Welfare, Dar es Salaam, Tanzania; 2 National Institute for Medical Research, Dar es Salaam, Tanzania; 3 Centre for Neglected Tropical Diseases (CNTD), Department of Parasitology, Liverpool School of Tropical Medicine, Liverpool, United Kingdom; 4 Department of Pathobiology and Diagnostic Investigation, Michigan State University, Michigan, United States of America; University of Zurich, SWITZERLAND

## Abstract

**Background:**

Lymphatic filariasis (LF) is best known for the disabling and disfiguring clinical conditions that infected patients can develop; providing care for these individuals is a major goal of the Global Programme to Eliminate LF. Methods of locating these patients, knowing their true number and thus providing care for them, remains a challenge for national medical systems, particularly when the endemic zone is a large urban area.

**Methodology/Principle findings:**

A health community-led door-to-door survey approach using the SMS reporting tool *MeasureSMS-Morbidity* was used to rapidly collate and monitor data on LF patients in real-time (location, sex, age, clinical condition) in Dar es Salaam, Tanzania. Each stage of the phased study carried out in the three urban districts of city consisted of a training period, a patient identification and reporting period, and a data verification period, with refinements to the system being made after each phase. A total of 6889 patients were reported (133.6 per 100,000 population), of which 4169 were reported to have hydrocoele (80.9 per 100,000), 2251 lymphoedema-elephantiasis (LE) (43.7 per 100,000) and 469 with both conditions (9.1 per 100,000). Kinondoni had the highest number of reported patients in absolute terms (2846, 138.9 per 100,000), followed by Temeke (2550, 157.3 per 100,000) and Ilala (1493, 100.5 per 100,000). The number of hydrocoele patients was almost twice that of LE in all three districts. Severe LE patients accounted for approximately a quarter (26.9%) of those reported, with the number of acute attacks increasing with reported LE severity (1.34 in mild cases, 1.78 in moderate cases, 2.52 in severe). Verification checks supported these findings.

**Conclusions/Significance:**

This system of identifying, recording and mapping patients affected by LF greatly assists in planning, locating and prioritising, as well as initiating, appropriate morbidity management and disability prevention (MMDP) activities. The approach is a feasible framework that could be used in other large urban environments in the LF endemic areas.

## Author summary

Lymphatic filariasis (LF) can cause disabling conditions in infected patients including lymphoedema-elephantiasis (LE) and hydrocoele. Identifying the number and locations of these patients is the first step towards ensuring that these patients receive the care they require, however there is currently no standardised approach for this essential action. This paper presents a health community-led approach for rapidly identifying patients in urban areas using an SMS reporting system, *MeasureSMS-Morbidity*, that allows health workers to report individual-level patient information (age, sex, location, condition, severity), which can be then be viewed in real-time via a web browser. The quality of the data can be easily monitored during the data collection period, and there is instant availability of patient information. This system is used here in the large urban centre of Dar es Salaam, Tanzania. A total of 6889 patients were identified, equating to 80.9 hydrocele patients per 100,000 population, 43.7 LE patients per 100,000 people, and 9.1 patients with both conditions. This information is now enabling the national neglected tropical disease (NTD) program to provide the essential care facilities and training for LF healthcare in locations in the city where it is most needed.

## Introduction

Lymphatic filariasis (LF) is a neglected tropical disease (NTD) that can have a devastating impact on affected individuals, with clinical symptoms such as acute dermatolymphangioadenitis (ADLA, “acute attacks”), lymphoedema and elephantiasis, and hydrocoele, causing physical, mental and economic distress [1–6]. In recognition of this, the World Health Organization’s (WHO) Global Programme to Eliminate Lymphatic Filariasis (GPELF) requires that countries wishing to be recognised as having eliminated LF are required not only to prove that disease transmission has been interrupted through mass drug administration (MDA), but that they are also alleviating the suffering of those affected by providing a minimum package of care to each person with lymphoedema/elephantiasis (LE) and hydrocoele in LF endemic areas [7]. This package includes (i) surgery for hydrocoele [8,9], (ii) support for episodes of ADLA [10,11], and (iii) management of LE to prevent disease progression. Countries wishing to complete the WHO dossier for certification of elimination as a public health problem are therefore required to firstly estimate the number of patients in endemic areas at the implementation unit (IU) level, and to provide information on the number of facilities able to provide the necessary care to these identified patients. Finally, an assessment of the readiness and quality of the care being provided at these services is also required [12,13].

Patient numbers at the IU level will enable national LF elimination programmes to appropriately forecast, plan and manage patient care, and to meet the requirements of the WHO dossier for programme success. At present, despite great progress towards interrupting transmission of disease with 63 of the 73 endemic countries having initiated MDA, only 18 of these are reported to monitor morbidity management and disability prevention (MMDP) at this geographical level, i.e. have identified the number of IUs with known cases, or the IUs where MMDP services are provided [12]. There are currently no specific guidelines on the methods for obtaining patient estimates, although an MMDP toolkit is currently under development by WHO to provide additional guidance for this. Current documented suggestions include collecting LE and hydrocoele information during LF baseline prevalence surveys, when enumerating households during MDA, or conducting separate surveys either independently or in collaboration with other organizations concerned with similar disabilities and their care [14]. Many countries are known to collate morbidity information whilst distributing MDA, however the quality of this information has been shown to be very variable, with national programmes lacking the necessary resources to validate their data [15,16]. Examples of bespoke patient enumeration surveys can also be found in the literature, however these tend to be on a small scale and are labour intensive and as such may be difficult to scale up to the required geographical level [16,17].

Dar es Salaam is a large and densely populated region on the coast of Tanzania which at the time of the survey (2015) comprised of three districts: Temeke, Kinondoni and Ilala. In this region LF is caused by the *Wuchereria bancrofti* parasite, transmitted by the *Culex* mosquito. Previous estimates of LF prevalence in Dar es Salaam measured using immunochromatographic tests (ICTs) includes 9.9% (Temeke = 13.2%, Kinondoni = 14.4%, Ilala = 4.7%), by Mwingira et al. (2017) [18], and 3.0% by Mwakitalu et al. (2013) [19]. Four rounds of MDA have been completed in the Dar es Salaam region since 2013, with varying therapeutic coverages due to the challenges associated with administering treatment within a large dynamic urban population. While it was anticipated that there was likely to be a significant number of LF clinical cases in Dar es Salaam, there was limited data on the scale of the problem. As such, there was a relative lack of evidence upon which to plan a suitable MMDP strategy [20].

The primary aim of this study was to improve our knowledge of the overall LF morbidity burden in Dar es Salaam, and further to gain an understanding of the geographical distribution of cases to guide the delivery of MMDP services to where they are most needed. Due to the association between the prevalence of clinical cases and of infection, knowing more about the locations of cases may also allow programmes to identify areas where MDA should be more focussed [21]. To undertake this task at this geographical scale in a time-effective manner, we implemented the mHealth short message service (SMS) reporting system, ‘*MeasureSMS-Morbidity’*, which enables the rapid collection, collation and dissemination of estimated LF patient numbers [22,23]. Prior to this study, *MeasureSMS-Morbidity* had been implemented in rural areas of Malawi and Ghana, but had yet to be trialled in a densely populated urban area [22]. The system relies upon a network of local health workers to each collect individual-level patient data over small geographical areas which they then submit via SMS using their own mobile phones. These data are then automatically collated into an online database which can be accessed and interrogated using a web browser. Mobile technology-driven, community-led approaches to conducting surveys such as this have proven to increase time efficiency, and consequently cost-efficiency, of data collection whilst further increasing the sense of local data ownership and accountability [23].

## Methods

### Ethics statement

LF patient identification and reporting activities are part of routine programme activities conducted by the Ministry of Health and Social Welfare, Tanzania, and as such ethical clearance in Tanzania was not required, and a waiver was granted. Oral consent to record and report details relating to their condition was obtained from all identified LF patients, however this was not documented. Reported and verified patients were verbally informed of the purpose of the activity, and all resulting data were analysed anonymously. Data reporters did not record any information on patients who refused to disclose any information, and those who did not wish to be examined by the clinical officer were excluded from the verification survey. Ethical clearance was obtained from the Liverpool School of Tropical Medicine Research Ethics Committee (Research Protocol 12.22).

### Patient identification survey

In order to achieve the primary aim of this study i.e. to assess the LF morbidity burden in Dar es Salaam, a patient identification survey was undertaken. This survey was undertaken in three phases between March and August 2015, covering each of Dar es Salaam’s three districts i.e. Temeke, Kinondoni and Ilala. To undertake this task efficiently with respect to both time and resources, a health community-led door-to-door survey approach was used and the *MeasureSMS-Morbidity* reporting tool was incorporated to rapidly collate and monitor the data. Detailed information on the *MeasureSMS-Morbidity* system can be found elsewhere [22–24]. In brief, this system allows basic information on identified patients such as their location, sex, and age, plus information on their clinical condition (LE or hydrocoele), severity of LE (mild, moderate, severe), and number of acute attacks experienced in the last 6 months, to be recorded by the health community staff and reported via SMS using their own phones. This information can then be viewed in real-time via a web browser. Each individual SMS report also generates a SMS response indicating either that the message had been sent in the correct format, or that there are reporting errors that required correcting. The severity of LE was classified as either mild = slight swelling, moderate = enlarged limb with shallow folds, and severe = greatly enlarges limb with deep folds as previously described [22]. These categories correspond to a Dreyer staging of 1–2 (mild), 3–4 (moderate) and 5+ (severe).

Prior to this current study, the *MeasureSMS-Morbidity* system had been implemented in rural settings only, and this was the first time at which the system had been implemented at such a large scale, and in an urban area. To facilitate this scale up, the implementation process was revised and refined after each phase. Each phase consisted of a training period, a patient identification and reporting period and a data verification period. This verification period was incorporated into the study to address the secondary objective of assessing data quality. Specifically, the verification period enabled the positive predictive value (PPV) of the identified patients to be estimated i.e. the proportion of patients identified during the survey who were confirmed to have lymphoedema or hydrocole. Phase 1 (Temeke) was initiated in March 2015, and Phases 2 (Kinondoni) and 3 (Ilala) was commenced in July and August 2015 respectively. The initial process and the subsequent refinements are described below.

### Training on LF morbidity and the SMS system

#### Initial process

**Fig 1:**
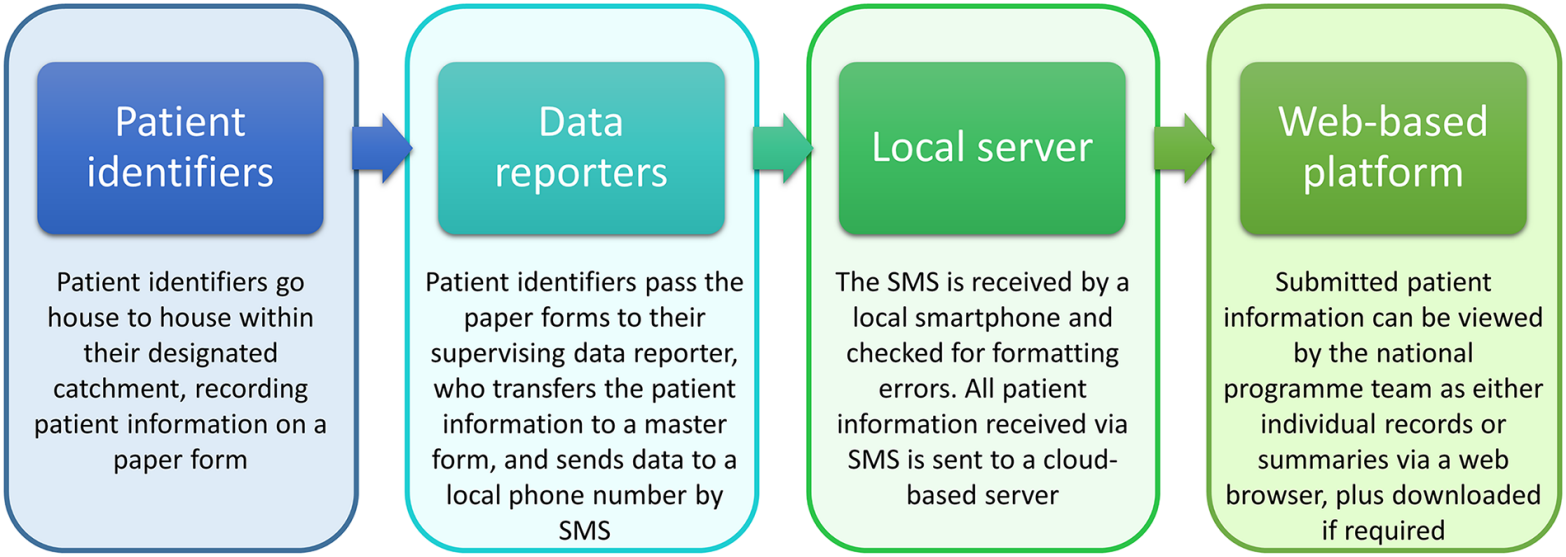
Flow of patient information during the patient identification exercise conducted in the three districts of Dar es Salaam.

Patient identification and reporting were undertaken using a two tiered approach [24]. Frontline health workers (one per facility) were invited to participate in the activity, during which they were to adopt the role of ‘data reporter’. Data reporters were required to: (1) train a team of ‘patient identifiers’, usually community health volunteers (CHVs), in how to identify and advise LE and hydrocoele patients, (2) supervise the patient identifier whilst they conducted a patient identification survey (3), collate the patient information that the patient identifiers record on paper forms at regular intervals throughout the survey period and (4) report this patient information by SMS using their own mobile phones, sending one SMS per identified patient (Fig 1).

Data reporters were provided with mobile phone credit for an allocated number of text messages for this purpose. The cost of sending an SMS varied according to the network used, and credit bundle purchased, with at most a single SMS costing $0.02 USD to send. To prepare them for the activity, the data reporters each attended a half day training session which focused on recognising and managing/treating LE and hydrocele (the precise information on a patient’s actual condition to be collected during the patient identification survey), how to use the paper-based data collection tools, and finally how to submit the relevant patient information by SMS using a simple but specific format. A practice exercise was undertaken during the training session to give each data reporter the opportunity to become familiar with the required format of the SMS under the supervision of the training team. The training sessions were also attended by a member of the district-level health team, whose role was to allocate patient identifiers to each data reporter and to support the overall supervision of the activity.

#### Refinements

The training process remained largely unchanged throughout the three phases of survey period. Greater emphasis was placed on common data reporting errors identified during previous phases, as well as the time allocated to practise sending data via SMS being increased, particularly for those who were less familiar with sending SMS messages. Further, in the second the third implementation phases, if a data reporter was identified as having extreme difficulty in sending the SMS during the practice sessions e.g. due to poor eyesight, a replacement data reporter for that facility was identified.

### Patient identification and reporting

#### Initial process

Patient identification was undertaken by CHVs, with each CHV being allocated a street (a “mtaa”) or part of a street within the vicinity of the health facility where their supervising data reporter was based. In general, these areas differed from those within which the CHV usually operated. CHVs were instructed to go door-to-door in their allocated area and identify individuals within a household with either clinical condition (LE or hydrocoele), and to record this information on a paper form. Identification was initially achieved by showing individuals illustrations of LE (mild, moderate and severe) and hydrocoele, and description using local terms for the conditions. Individuals would then self-report these conditions, after which the CHV would confirm LE and severity through examination; hydrocoele cases were not confirmed through examination. Following this, the CHV would give basic MMDP advice to patients. At the end of each day this paper form was given to their designated data reporter, who transferred the information to a master form, and sent the information in via SMS. It is during this phase that each identified patient was allocated a unique identification number which was used to link the SMS record back to the patient, as patient names were not contained in the SMS. These SMS reports were monitored in real-time by the supervising team via the web browser, allowing any reporting errors or inconsistencies to be identified and rectified.

#### Refinements

The data collection forms were amended following the first implementation phase to include the patient’s colloquial or ‘famous’ name, and both the patient’s and the patient identifier’s phone numbers. This information greatly facilitated patient follow-up as prior to this follow-up was only possible via the patient identifier. The monitoring process was also adapted after the first iteration such that clarification was sought if a patient’s age was reported to be less than 10 years, or if a patient was reported to have both conditions.

### Verification of reported patients

#### Initial process

To assess the quality of the patient identification data, particularly the accuracy of the reported clinical condition, a follow-up verification survey was conducted after each patient identification period. The verification team consisted of a clinical officer plus the original patient identifier where possible, to ensure consistency for the patient. During this process, patients were provided with additional advice on how to manage their condition and/or access treatment.

The process used to select patients to verify during the first phase was similar to that used in previous small-scale rural surveys conducted in Malawi and Ghana, i.e. a (stratified) random sample of patients were selected and visited by a LF morbidity expert to assess the presence and severity of the clinical condition. Additional demographic information was also collected for comparison against that which was reported by SMS. The survey team was provided with the unique patient ID and the reported sex and condition of the patient only to allow the remaining patient information (age, severity of condition, number of acute attacks experienced in the last 6 months) to be collected independently from the original SMS report. Verification survey sample size calculations were based on the expected positive predictive value (PPV) i.e. the proportion of identified patients who were confirmed to have their reported condition [22]. Based on previous surveys we assumed a PPV of 0.9 for lymphoedema and 0.8 for hydrocoele, and the sample size calculation was based on a 95% confidence level and 10% precision. On completion of the verification survey, the data recorded by the verification team were compared with that reported by SMS during the patient identification survey. The PPV for each condition, and overall PPV were then calculated.

#### Refinements

The initial verification process was determined to be too time consuming and labour intensive in a densely populated urban environment. Whilst information on the area in which the patient resided was collected by the patient identifiers, and recorded on the data reporter’s initial master form, the exact location of the patient was difficult to pinpoint without the assistance of the patient identifier. As the patient identifier was not always available during the verification period, this slowed down the patient locating process. Furthermore, following the first phase of patient identification in Temeke, Dar es Salaam was subject to severe flooding which resulted in difficulties in accessing targeted areas plus population displacement. The addition of the identified patient’s phone number in the data collection sheet was recognised as one way to alleviate these difficulties. Further, a revised sampling strategy was also adopted for Phases 2 (Kinondoni) and 3 (Ilala) i.e. a cluster-based sampling strategy using the data reporters as clusters. As data reporters were responsible for training the patient identifiers in recognising LE and hydrocoele it was recognised that there would potentially be between-cluster variation in PPV, which needed to be accounted for when calculating the required sample size for a cluster-based verification survey. However, at this time there were no data available to explore this further and obtain an estimate of the design effect, a conservative approach was therefore taken, i.e. the sample size was increased by 10% to account for a moderate amount of between cluster variability. Data reporters were selected randomly, and it was then the data reporters’ responsibility to select a fixed number of patients from their list to be examined.

#### Analysis

Summaries of the cases reported by SMS were produced including the number and overall incidence of cases by district, age, sex, and severity. Further, ward-level maps of the reported data were produced, and the mean number of acute attacks within the last 6 months was explored by severity was calculated. The quality of the reported data was assessed via summaries of the percentage of correctly submitted SMS messages by district over time and between districts. The significance of the differences in district-level acceptance rates were assessed using a difference in proportions test. PPV summaries of the verification data were produced, and Cohen’s kappa was used to determine the level of agreement in the severity of condition as assessed by the patient identifiers and the verification team.

## Results

### Reported data

Table 1 presents the number of patients with each condition reported by SMS. In absolute terms, Kinondoni had the highest number of reported patients (2846) comprised of 950 LE only patients, 1654 hydrocoele only patients, and 242 with both conditions. This was followed by Temeke (2550 total, 807 LE, 1560 hydrocoele, 183 with both), then Ilala (1493 total, 494 LE, 955 hydrocoele, 44 with both).

**Table 1:** Reported lymphoedema-elephantiasis and hydrocoele prevalence for each of Dar es Salaam’s three districts.

	Lymphoedema-Elephantiasis only		Hydrocoele only		Both		Total	
	N	Prevalence per 100,000 total population	N	Prevalence per 100,000 total population (per 100,000 male population)	N	Prevalence per 100,000 total population	N	Prevalence per 100,000 total population
**Temeke (Phase 1)**	807	49.8	1560	96.2 (220.4)	183	11.3	2550	157.3
**Kinondoni (Phase 2)**	950	46.4	1654	80.7 (183.2)	242	11.8	2846	138.9
**Ilala (Phase 3)**	494	33.3	955	64.3 (150.5)	44	3.0	1493	100.5
**Total**	**2251**	**43.7**	**4169**	**80.9 (185.7)**	**469**	**9.1**	**6889**	**133.6**

Prevalence estimates (per total population and per male population for hydrocoele) were calculated using the 2015 population estimates as the denominator, which were extrapolated from the 2012 census using annual growth rates of 5.8%, 4.9% and 6.5% for Temeke, Kinondoni and Ilala respectively, i.e. population estimates of 1,621,148, 2,048,976 and 1,485,308, totalling 5,155,432, with male population estimates of 707,861, 902,981 and 634,663 totalling 2,245,505 [25]. The overall reported morbidity prevalence for Dar es Salaam was 133.6 per 100,000 total population, with the highest prevalence seen in Temeke (157.3 per 100,000) and the lowest in Ilala (100.5 per 100,000). This pattern was consistent for both conditions, with LE only prevalence per 100,000 population ranging from 33.3 in Ilala to 49.8 in Temeke, and hydrocoele only prevalence per 100,000 males ranging from 150.3 in Ilala to 220.4 in Temeke. The ratio of LE to hydrocoele patients was consistent across all three districts, with the number of hydrocoele patients being almost twice that of LE cases.

Table 2 presents summaries of the reported patients by age and sex. District-level population data was obtained from the National Bureau of Statistics [26] for 2012, and the totals were projected to represent 2015 as described above. The prevalence of LE was approximately equal between males and females in all three districts (Temeke: 64.84 and 71.72 per 100,000 population; Kinondoni: 67.89 and 60.37; Ilala 39.86 and 42.84 for males and females, respectively). A positive relationship was observed between age and LE prevalence in all three districts and both sexes, with the highest prevalence being observed in the oldest age group in Temeke (>74 years, 708.44 patients per 100,000 population), and in the 60–74 age group in Kinondoni (474.30 patients per 100,000 population) and Ilala (353.16 per 100,000 population). For hydrocoele, a similar trend in age was observed (1,075.4 patients per 100,000 population aged >74 in Temeke, 727.8 patients per 100,000 population aged 60–74 in Kinondoni and 614.2 patients per 100,000 population aged 60–74 in Ilala).

**Table 2:** Demographic summaries of reported patients.

		Lymphoedema-Elephantiasis (include both conditions)						Hydrocoele	
		Male		Female		Total		Male Total	
		N	Prevalence per 100,000 total population	N	Prevalence per 100,000 total population	N	Prevalence per 100,000 total population	N	Prevalence per 100,000 total population (per 100,000 males)
**Temeke**	**0–14**	1	0.42	5	2.05	6	1.25	23	4.8 (9.7)
	**15–29**	37	16.39	66	24.40	103	20.76	124	25.0 (54.9)
	**30–44**	116	73.41	167	111.15	283	91.80	532	172.6 (336.7)
	**45–59**	138	226.85	148	285.56	286	253.86	521	462.4 (856.4)
	**60–74**	113	534.94	104	618.50	217	571.97	408	1075.4 (1931.4)
	**>74**	52	917.48	39	543.37	91	708.44	131	1019.8 (2311.3)
	**NA**	2	-	2		4		4	
	**Total**	**459**	**64.84**	**531**	**71.72**	**990**	**68.36**	**1743**	**120.3 (246.2)**
**Kinondoni**	**0–14**	18	6.58	6	2.12	24	4.31	57	10.2 (20.8)
	**15–29**	66	21.74	52	13.69	118	17.27	165	24.1 (54.3)
	**30–44**	206	95.25	204	102.19	410	98.58	635	152.7 (293.6)
	**45–59**	171	224.21	177	263.27	348	242.51	593	413.2 (777.5)
	**60–74**	122	451.83	110	502.00	232	474.30	356	727.8 (1318.5)
	**>74**	29	474.44	30	400.88	59	433.95	88	647.2 (1439.7)
	**NA**	1	-	0	-	1		2	-
	**Total**	**613**	**67.89**	**579**	**60.37**	**1192**	**64.02**	**1896**	**101.8 (210.0)**
**Ilala**	**0–14**	3	1.45	1	0.47	4	0.96	25	6.0 (12.1)
	**15–29**	27	13.42	37	15.04	64	14.31	57	12.7 (28.3)
	**30–44**	74	50.39	71	52.08	145	51.20	273	96.4 (185.9)
	**45–59**	79	141.35	89	186.31	168	162.07	365	352.1 (653.1)
	**60–74**	57	298.15	68	415.20	125	352.16	218	614.2 (1140.3)
	**>74**	13	259.71	19	288.07	32	275.84	61	525.8 (1218.7)
	**Total**	**253**	**39.86**	**285**	**42.84**	**538**	**41.39**	**999**	**76.8 (157.4)**
**Total**	**0–14**	22	3.07	12	1.62	34	2.34	105	7.2 (14.6)
	**15–29**	130	17.80	155	17.29	285	17.52	346	21.3 (47.4)
	**30–44**	396	75.98	442	90.90	838	83.19	1440	142.9 (276.3)
	**45–59**	388	201.05	414	248.16	802	222.89	1479	411.0 (766.4)
	**60–74**	292	434.25	282	511.75	574	469.15	982	802.6 (1460.4)
	**>74**	94	560.00	88	413.99	182	478.41	280	736.0 (1668.1)
	**NA**	3		2		5	-	6	-
	**Total**	**1325**	**59.01**	**1395**	**58.99**	**2720**	**59.00**	**4638**	**100.6 (206.5)**

Table 3 presents summaries of severity of reported LE (mild, moderate, severe) by district, including the mean and standard deviation of the number of reported number of acute attacks experienced in the previous six months. Overall, severe LE patients account for approximately a quarter of those reported (26.9% overall ranging from 25.0% in Temeke to 31.0% in Ilala), with the number of acute attacks increasing with reported severity (1.34 in mild cases, 1.78 in moderate cases, 2.52 in severe). The variability in the number of reported attacks also increases with severity (1.33 in mild cases, 1.62 in moderate, 1.84 in severe). Similar trends are seen in each of the three districts.

**Table 3:** Reported LE severity and acute attacks summary.

		Mild (stage 1–2)	Moderate (stage 3–4)	Severe (stage 5+)	Total
**Temeke**	**N**	319	260	193	772
	**% of patients**	41.3%	33.7%	25.0%	100%
	**Mean attacks**	1.67	2.06	2.83	1.75
	**SD attacks**	1.51	2.00	2.00	1.89
**Kinondoni**	**N**	350	325	244	919
	**% of patients**	38.1%	36.5%	26.6%	100%
	**Mean attacks**	1.16	1.71	2.39	1.51
	**SD attacks**	1.08	1.15	1.72	1.50
**Ilala**	**N**	106	208	141	455
	**% of patients**	23.3%	45.7%	31.0%	100%
	**Mean attacks**	0.99	1.53	2.30	1.45
	**SD attacks**	1.28	1.65	1.78	1.65
**Total**	**N**	775	793	578	2146
	**% of patients**	36.1%	37.0%	26.9%	100%
	**Mean attacks**	1.34	1.78	2.52	1.58
	**SD attacks**	1.33	1.62	1.84	1.68

**Fig 2:**
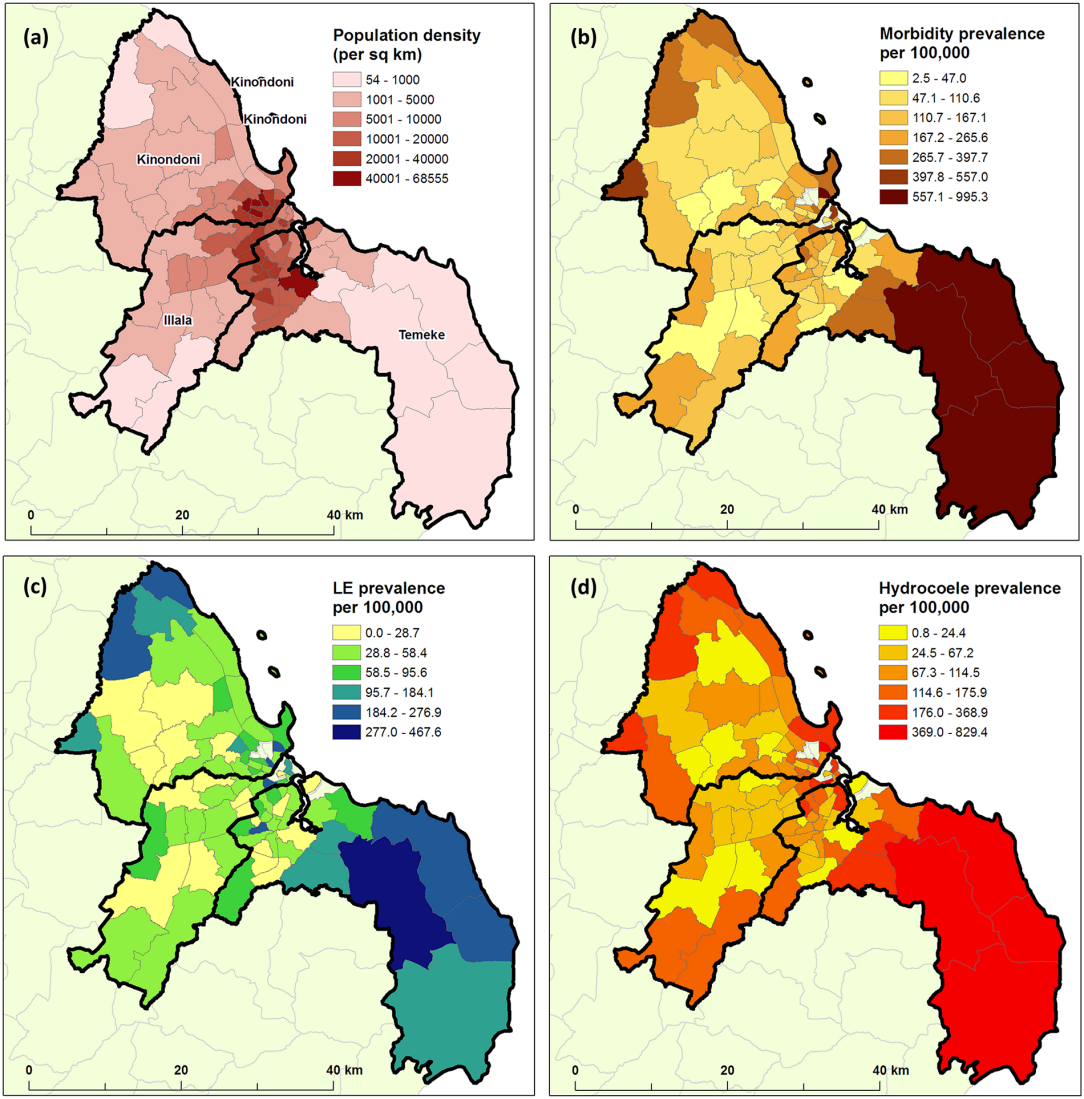
(a) Population density at the ward level for 2015 using extrapolated data from 2012 census, and the reported morbidity prevalence per 100,000 people obtained using the MeasureSMS-Morbidity system (b) overall,(c) for LE and (d) for hydrocoele.

Maps of prevalence by ward level, using extrapolated 2015 population estimates as the denominator (http://ihi.eprints.org/2168/1/Village_Statistics.pdf) are presented in Fig 2. These figures highlight the high morbidity prevalence in the southern peri-urban wards of Temeke and the northern peri-urban wards of Kinondoni.

### Accuracy of data

**Fig 3:**
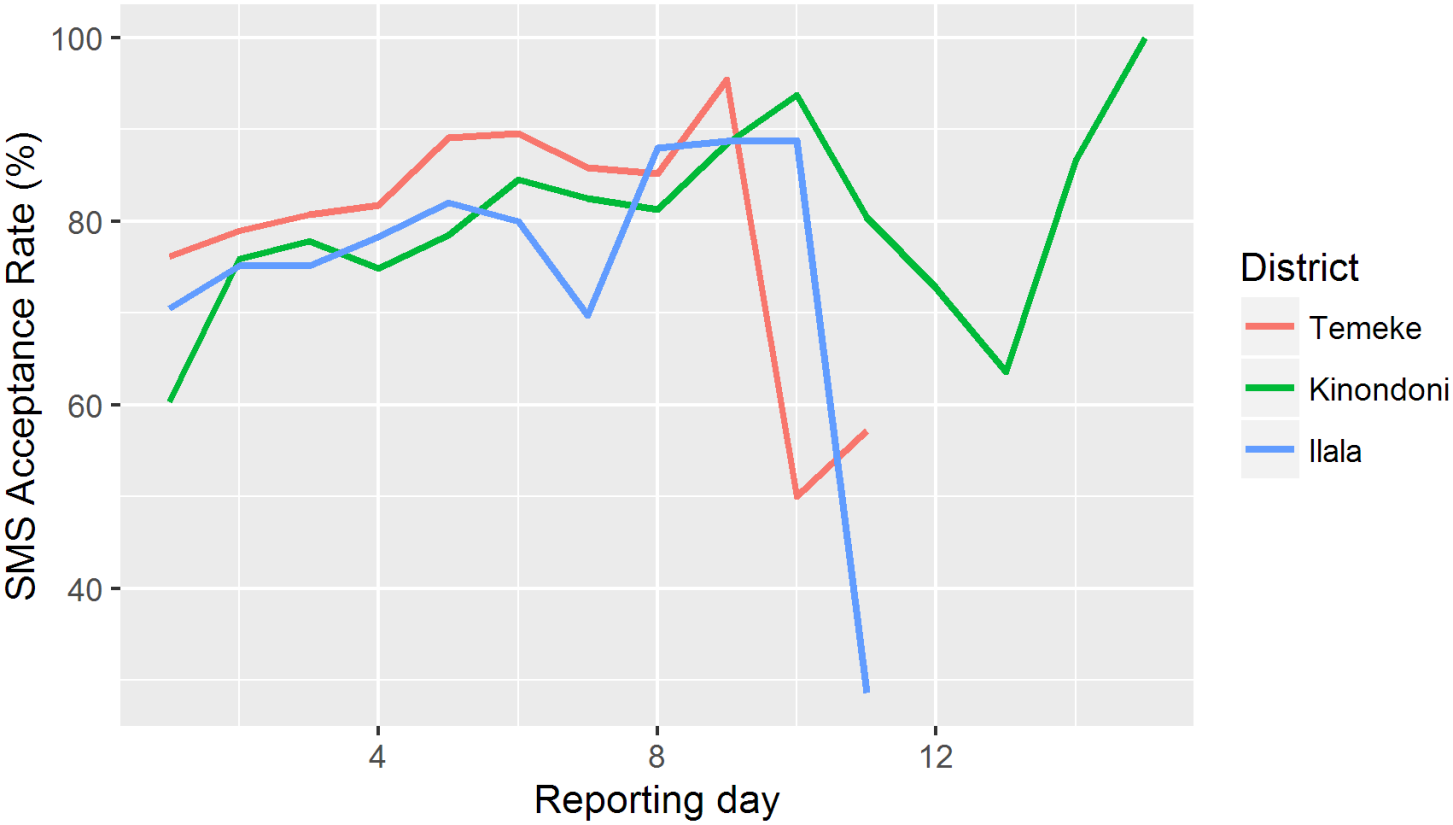
SMS acceptance rates over the patient identification period by day of reporting for each district.

The *MeasureSMS-Morbidity* system stores every SMS received and automatically checks the SMS for formatting errors. Table 4 summarises the SMS acceptance rates (% of SMS without any formatting errors) for each of the three patient identification phases. These error rates reflect two processes. Firstly, they give an indication of the data reporters’ ability to report data using the *MeasureSMS-Morbidity* system, which may in turn reflect improvements in the training process. Secondly, they reflect the effectiveness of the data supervision process, as the incoming data were reviewed each day by the supervision team and data reporters were contacted when repeated errors or data inconsistencies were identified. During Phase 1 (Temeke), data supervision was largely undertaken by the donors whereas phases 2 (Kinondoni) and 3 (Ilala) was managed by the Ministry of Health staff, with support from the donor. In comparing the acceptance rates between these three period, no significant differenced were observed (p = 0.3788). However once duplicates were removed, significant differences were observed (p<0.001), with a greater proportion of accepted messages being kept in phase 1 (Temeke = 98.5%) in comparison to phases 2 and 3 (Kinondoni = 94.7 and Ilala = 94.0%). Fig 3 presents the acceptance rate by reporting day for each of the three districts. We again observe slightly higher acceptance rates by day in Phase 1 (Temeke). Table 4 indicates that in each district, data were reported over a period of 12–15 days, with most data (greater than 80%) being sent in the first 7 days of the reporting period. We therefore hypothesise that the decline in acceptance rates towards the end of the patient identification period seen in Fig 3 is likely due to there only being a small number of reports being submitted during this time, with these data reporters being those who had difficulties using the system.

**Table 4:** Summary of messages received during the patient identification period.

	Temeke		Kinondoni		Ilala	
	N	%	N	%	N	%
**Number of data reporters**	118		148		92	
**Approximate number of patient identifiers**	600		500		400	
**Number of reporting days**	12	-	15		12	
**SMS received**	3126	-	3594		1885	-
**SMS received in first 7 days**	2980	95.3%	2945	82.9%	1527	81.0%
**SMS accepted with no formatting errors**	2588	82.8%	3006	83.6%	1588	84.2%
**Cleaned SMS reports (after removing duplicates etc.)**	2550	98.5%	2846	94.7%	1493	94.0%

The length of the verification surveys varied with each phase. Due to adverse flooding conditions experienced in March and May 2015, the simple (stratified) random sampling approach used, and the difficulties in locating selected patients as described in the methods section, the verification process in Temeke lasted over 13 months and was completed in May 2016. Over this period, 38 patients were randomly selected for verification and visited by the verification team. No issues were observed between paper forms and data sent via SMS when paper forms were reviewed as the initial part of the verification. When comparing the written records collected during this verification visit with the initial data reported during the patient identification survey it was possible to match 36 (15 reported LE, 21 reported hydrocoele) of the 38 patients by patient ID and gender. Of these 36, the main difference observed were in the initially reported age and the age reported during verification, with a median difference of two years. As inconsistencies in age recall is common in these settings, differences of up to 10–15 years were expected. Four matched patients in Temeke had an age difference of greater than 15 years.

Adaptations made to the data collected during patient identification i.e. the inclusion of the patient’s phone number, plus the refinements made to the sampling process resulted in the verification for Kinondoni and Ilala being completed much more efficiently. There were however still some delays due to the transience of the population in Dar es Salaam as well as some restructuring of the city resulting in large areas of houses being demolished between the time of patient identification and data verification period. In both Kinondoni and Ilala, 115 patients, reported by 16 data reporters in each district, were verified over a 6 month period. Of the 115 patients in Kinondoni it was not possible to match 17 patients with the reported data, hence only 98 verified patients (24 reported LE, 71 reported hydrocoele, 3 reported with both conditions) were included in the final dataset. The median difference in reported age for these 98 patients was two years, with 10 patients having age differences of greater than 15 years. In Ilala, only one patient could not be matched, hence 114 patients were included (30 reported LE, 75 reported hydrocoele, 9 reported with both conditions). The median difference in reported age of these patients was three years, with 12 patients having age differences greater than 15 years.

Table 5 presents the verification results by district. Of the 81 patients reported to have either LE or both conditions, 63 (77.8%) were diagnosed by the verification team as having LE, ranging from 71.8% (28/39) in Ilala to 86.7% (13/15) in Temeke. Of the 179 patients reported to have hydrocoele or both conditions, 165 (92.2%) were diagnosed as having hydrocoele by the verification team, ranging from 88.1% (74/84) in Ilala to 95.9% (71/74) in Kinondoni. Similar results were observed after removing the 26 patients whose reported age differed by more than 15 years between the two data sources (S1 Table).

**Table 5:** Verification data summaries. Note that the reported lymphoedema-elephantiasis and hydrocoele rows include those who were reported to have both conditions. The report was considered to be correct if the patient was confirmed to have the reported condition, regardless of whether or not another LF-related condition was also reported or confirmed.

	Temeke	Kinondoni	Ilala	Total
**Lymphoedema-Elephantiasis**				
Reported	15	27	39	81
Correct	13	22	28	63
% Correct	86.7%	81.5%	71.8%	77.8%
**Hydroceoele**				
Reported	21	74	84	179
Correct	20	71	74	165
% Correct	95.2%	95.9%	88.1%	92.2%

Of the 18 people misdiagnosed with LE, 14 were confirmed to have hydrocoele, whereas 4 had other conditions. Of the 14 people misdiagnosed with hydrocoele, 5 had LE, 1 had a hernia, 2 had previously had hydrocoele but had been operated on, 5 had other conditions and 1 did not have any discernible illness. It is also worth noting that of the 165 confirmed to have hydrocoele, 27 (16.3%) had a concurrent hernia. Twelve patients with both LE and hydrocoele were included in the verification survey. Only 4 (33.3%) of these were confirmed to have both conditions, 5 had hydrocoele only, 2 had LE only and 1 had a non-LF associated condition.

Of the 63 patients correctly verified as having LE, 60 had reported the severity of the condition (11 mild, 26 moderate, 23 severe). In comparing these with the Dreyer score determined for each patient during the verification process (stage 1–2 = mild, stage 3–4 = moderate, stage 5+ = severe), 3 (27.3%) were correctly verified as mild, 16 (61.5%) were correctly identified as moderate and 6 (26.1%) were correctly identified as severe (Table 6). Overall, there was low agreement between the severity assigned by the patient identifiers and the verification team (Cohen’s kappa = 0.083).

**Table 6:** A comparison of reported and verified lymphoedema-elephantiasis severity.

		**Verified**			
		**Mild**	**Moderate**	**Severe**	**Total**
**Reported**	**Mild**	3	8	0	11
	**Moderate**	8	16	2	26
	**Severe**	4	13	6	23
	**Total**	15	37	8	60

## Discussion

### Morbidity burden

With the prevalence of LF cases previously unrecorded, these results indicate a much higher burden of LE and hydrocoele in Dar es Salaam than anticipated for an urban centre, with 2251 patients reported to have LE, 4169 patients reported to have hydrocoele plus a further 469 patients having both conditions. Whilst it is recognised that there may be some false positives in these reports, and further that some patients may have been missed from the survey, the verification survey results give the national LF elimination programme confidence that these numbers are representative of the true burden. Further to these absolute numbers, the national programme now also has additional information on the geographical distribution of disease, with the district of Temeke being the most affected of Dar es Salaam’s three districts with the larger, less densely populated wards reporting in excess of 500 cases per 100,000 people.

This information is crucial in determining the resources needed to manage the conditions affecting these patients, and will assist in selection in locations within the urban area within which care services could be most effectively provided. As with many endemic countries, strategic partnerships have been developed in Tanzania between the national LF programme and international donors to address the immense burden of LF clinical disease within the country, leading to the development of plans to scale up hydrocele surgery throughout 2016. Further, plans to engage CHVs to provide home-based LE training and care for patients and their caregivers have already been developed with the region’s NTD and home-based care teams. These services are intended to be supplementary to the specialist filariasis clinic which has been operated by the national programme in Dar es Salaam since 2000 [20].

The data obtained in this study will also, in addition to guiding morbidity management activities, provide an insight into disease transmission within Dar es Salaam which is likely to aid the management of future MDA campaigns, or perhaps the development of alternative transmission breaking strategies such as vector control through improved drainage and *Culex* mosquito population reduction e.g. using polystyrene beads [27,28]. Difficulties in conducting MDA within an urban environment often arise due to the factors such as high population movement, and the resulting low compliance can result in MDA coverage levels that are inadequate to permanently break transmission. It is also not clear to what extent the distribution of patients reflects the transmission patterns in such a mobile urban population. However, the additional disease transmission information provided by patient mapping may be useful in raising awareness across the city in general, targeting potential high risk areas with more intensive community sensitisation campaigns, and/or increasing MDA distribution points to increase coverage [18,19,29,30]. It is likely that future MDAs will benefit from the active and successful morbidity management activities, as it has been shown that LE management programmes can be important for improving increasing MDA coverage [31].

### mHealth system for patient identification

In implementing the two-tiered patient identification and reporting process in an iterative manner, it was possible to refine the process with each phase of implementation. Relocating patients after the initial patient identification survey proved to be the most challenging aspect of this approach, with unavoidable contributing factors including adverse weather conditions and population displacement due to the demolition of many houses. Difficulties also arose due to the patient identifier being unfamiliar with the area to which they had been assigned. Whilst this problem lessened over the course of the exercise, primarily through ensuring the national programme could contact the patient directly by mobile phone, it may be beneficial to consider using locally-based patient identifiers should this exercise be repeated elsewhere. Additionally, by increasing the emphasis on the staging and severity of LE in the training, the accuracy of the reported data could be improved, thus providing more accurate information on the level of services required in any given area.

Despite the implementation challenges, there were many benefits to the patient identification and the reporting process adopted for this exercise. Notably, the ability to view and assess the quality of the patient identification data in real time using the *MeasureSMS-Morbidity* tool was a great asset. Enabling information on the morbidity burden to be known almost instantaneously, as opposed to having to wait a prolonged period for collation and digitization of the paper forms, also facilitates the provision of needed care to patients much more efficiently [20]. By the end of the study, the responsibility of monitoring the real-time data and ensuring its quality was led by the national programme team themselves, thus empowering the local teams to manage and control their own success. This approach of promoting the national programme to take full ownership of the data and the implementation of the process had the effect of building valuable data management and health surveillance capacity within the local team [32,33].

This exercise highlights how crucial patient estimates are for the provision of much needed care to LF patients and the information obtained greatly assists in planning, locating and prioritising, as well and initiating, appropriate MMDP activities. The approach to patient identification and reporting presented in this paper provide a feasible framework that could be adopted in other large urban environments [24], and thereby enable these endemic areas to achieve the MMDP components of the GPELF [12], or could be adapted to address patient identification issues for other diseases. The focus should now be to develop and implement strategies to meet the needs of the patients identified during this exercise, and thus ensuring no patient is left behind.

## Supporting information

S1 Table Verification data summaries, excluding patients with a reported age difference greater than 15 years. Note that the reported lymphoedema-elephantiasis and hydrocoele rows include those who were reported to have both conditions. The report was considered to be correct if the patient was confirmed to have the reported condition, regardless of whether or not another LF-related condition was also reported or confirmed.
(DOCX)
