# Supplementary material for: Lymphatic filariasis patient identification in a large urban area of Tanzania: An application of a community-led mHealth system
Source: PLoS Negl Trop Dis. 2017 Jul 14;11(7):e0005748. doi: 10.1371/journal.pntd.0005748 (PMC5529014; doi:10.1371/journal.pntd.0005748)
Supplement: S1 Table — Note that the reported lymphoedema-elephantiasis and hydrocoele rows include those who were reported to have both conditions. The report was considered to be correct if the patient was confirmed to have the reported condition, regardless of whether or not another LF-related condition was also reported or confirmed. (DOCX) [file pntd.0005748.s001.docx]

|  | **Temeke** | **Kinondoni** | **Ilala** | **Total** |
| --- | --- | --- | --- | --- |
| **Lymphoedema-Elephantiasis** |  |  |  |  |
| Reported | 14 | 23 | 34 | 71 |
| Correct | 13 | 20 | 25 | 58 |
| % Correct | 92.9% | 87.0% | 73.5% | 81.7% |
|  |  |  |  |  |
| **Hydroceoele** |  |  |  |  |
| Reported | 18 | 68 | 75 | 161 |
| Correct | 17 | 66 | 69 | 152 |
| % Correct | 94.4% | 97.1% | 92.0% | 94.4% |

*S1 Table: Verification data summaries, excluding patients with a reported age difference greater than 15 years. Note that the reported lymphoedema-elephantiasis and hydrocoele rows include those who were reported to have both conditions. The report was considered to be correct if the patient was confirmed to have the reported condition, regardless of whether or not another LF-related condition was also reported or confirmed.*
